# Research progress in the pathogenesis of hormone-induced femoral head necrosis based on microvessels: a systematic review

**DOI:** 10.1186/s13018-024-04748-2

**Published:** 2024-04-26

**Authors:** Tiancheng Ma, Yan Wang, Jianxiong Ma, Hongwei Cui, Xiaotian Feng, Xinlong Ma

**Affiliations:** 1 Tianjin Hospital of Tianjin University, Tianjin, 300211 China; 2 Tianjin Orthopedic Institute, Tianjin, 300050 China; 3 Tianjin Key Laboratory of Orthopedic Biomechanics and Medical Engineering, Tianjin, 300050 China

**Keywords:** Osteonecrosis of the femoral head, Glucocorticoids, Angiogene-osteogenesis coupling, H-type vessels, HIF-1α, PDGF-BB, VGEF

## Abstract

Hormonal necrosis of the femoral head is caused by long-term use of glucocorticoids and other causes of abnormal bone metabolism, lipid metabolism imbalance and blood microcirculation disorders in the femoral head, resulting in bone trabecular fracture, bone tissue necrosis collapse, and hip dysfunction. It is the most common type of non-traumatic necrosis of the femoral head, and its pathogenesis is complex, while impaired blood circulation is considered to be the key to its occurrence. There are a large number of microvessels in the femoral head, among which H-type vessels play a decisive role in the “angiogenesis and osteogenesis coupling”, and thus have an important impact on the occurrence and development of femoral head necrosis. Glucocorticoids can cause blood flow injury of the femoral head mainly through coagulation dysfunction, endothelial dysfunction and impaired angiogenesis. Glucocorticoids may inhibit the formation of H-type vessels by reducing the expression of HIF-1α, PDGF-BB, VGEF and other factors, thus causing damage to the “angiogenesis-osteogenesis coupling” and reducing the ability of necrosis reconstruction and repair of the femoral head. Leads to the occurrence of hormonal femoral head necrosis. Therefore, this paper reviewed the progress in the study of the mechanism of hormone-induced femoral head necrosis based on microvascular blood flow at home and abroad, hoping to provide new ideas for the study of the mechanism of femoral head necrosis and provide references for clinical treatment of femoral head necrosis.

## Introduction

Ascular necrosis of the Femoral Head (ONFH), also known as avascular necrosis of the femoral head (ANFH), occurs when blood flow to the femoral head is interrupted, It leads to the lack of support of the head and apoptosis, and then compensatory repair of the human body, resulting in the loss of femoral head cartilage, bone collapse and other diseases [1]. The causes of ischemia can be divided into arterial obstruction and venous stasis [2]. Many scholars have put forward a large number of theories on the mechanism of femoral head necrosis, including blood circulation disorders, lipid metabolism disorders, increased internal bone pressure, osteocyte apoptosis, gene polymorphism, immune factors, biomechanical theories of femoral head [3] etc., but the specific pathogenesis is still unclear, and it is often caused by multiple factors, and various theories explain and discuss its pathogenesis from different levels.

Femoral head necrosis tends to occur in young and middle-aged people and tends to be younger. Because of its potential occurrence, it often leads to disability and brings great distress to patients and families [4, 5]. Although increasingly sophisticated surgical techniques such as joint replacement have improved the quality of life for many patients, total hip replacement is not an optimal treatment option for ONFH due to the limited implant life and potential revision complications. How to treat early ONFH, prevent disease progression and delay the collapse time of femoral head necrosis is still puzzling many scholars [6–8]. Currently, hip preservation operations widely used mainly include core decompression [9], core decompression combined with bone graft with or without vascular pedicle [10, 11], core decompression combined with porous material support [12], core decompression combined with stem cell transplantation [13], osteotomy [14], etc. However, the clinical efficacy of these methods is not uniform [15], and the treatment prognosis is affected by many factors [16]. Still, a large proportion of ONFH may develop into femoral head collapse, which eventually leads to loss of hip function and necessitates joint replacement. Therefore, it is of great significance to explore the mechanism of femoral head necrosis and find more perfect treatment. Currently, femoral head necrosis is divided into two categories. The first type is caused by trauma, which is mainly caused by femoral neck fracture, hip dislocation and other diseases. The second type is non-traumatic and is caused by a synergistic combination of genetics and exposure to risk factors, including steroid use or alcohol abuse. However, according to the "ischemia hypothesis", the direct cause is the decrease or interruption of the blood supply in the femoral head, and the normal metabolism can’t cause necrosis. Hormone is the most common cause of non-traumatic femoral head necrosis, and it has been reported that hormonal femoral head necrosis has become the most common type of non-traumatic femoral head necrosis, accounting for up to 51% [17]. Weinstein et al. showed that 30–50% of patients with long-term use of GCs may have fractures, and another 5–40% of patients may have osteonecrosis [18]. In the United States, more than 30 million patients require glucocorticoids for disease treatment, and more than 40 percent will develop osteonecrosis [19]. Glucocorticoids (GCs) are widely used in the treatment of inflammation, autoimmune diseases, cancer and other diseases [20]. Long-term use of GCs has serious effects on stem cell differentiation, lipid metabolism and vascular system.

Femoral head blood flow injury is an important cause of femoral head necrosis. There are a large number of microvessels in the femoral head, especially H-type vessels, which are the main vessels that determine osteogenesis and bone reconstruction of the femoral head [21]. In recent years, more and more studies have shown that glucocorticoids damage the vaso-bone remodeling coupling through the vascular system, especially by inhibiting the formation of H-type blood vessels, leading to a serious decline in bone reconstruction and repair ability, and promoting the progression of femoral head necrosis. Based on the mechanism of glucocorticoids' influence on microvascular blood flow of femoral head, this review reviewed domestic and foreign studies to clarify the close relationship between microangiogenesis, bone reconstruction and collapse of femoral head necrosis in hormone-induced femoral head necrosis, in order to provide reference for the study of hormone-induced femoral head necrosis and provide theoretical basis for inhibiting clinical treatment of femoral head necrosis.

## Vaso-osteogenic coupling

The process of bone growth and development involves bone modeling and bone remodeling. In bone modeling, bone formation occurs independently of bone resorption, while in the process of bone remodeling, bone resorption and bone formation are coupled to maintain bone homeostasis [22]. The imbalance between bone resorption and bone formation will lead to the occurrence of osteoporosis, bone necrosis, fracture malunion and other diseases [23]. Angiogenesis, the development of new blood vessels from pre-existing blood vessels, is closely related to bone development and bone formation during bone modeling and remodeling. Blood vessels not only provide the necessary nutrients, oxygen, growth factors and hormones for bone tissue, in recent years, studies have found that blood vessels play a crucial role in the regulation of bone formation [24].

Although there are differences in the association of bone formation and bone resorption during osteogenesis and bone remodeling, both processes are combined with angiogenesis [25].The close spatial and temporal connection between osteogenesis and angiogenesis is known as the "angiogene-osteogenesis coupling" [26].In a mouse model of osteoporosis after ovariectomy (OVX), Wang [27] administered PEMF with specific parameters 12 weeks after surgery and continued for 8 weeks. The results showed that PEMF could effectively counteract OVX-induced bone loss, which was characterized by increased trabecular bone and increased transcription expression of Osterix, PDGFB and Col-1a1. Regulation of bone anabolic and catabolic activity. CD31 and Endomucin double positive immunostaining and flow cytometry showed that PEMF-induced osteogenesis was associated with CD31-endothelial cell expansion. At the same time, HIF-α levels were higher in PEMF-treated mice than in control mice, while inhibition of HIF-1α significantly reduced PEMF-induced osteogenesis and led to a significant reduction of CD31hiEmcnhi vessels in PEMF-treated OVX mice. This study confirmed the coupled promotion of PEMF-induced bone formation and CD31hiEmcnhi endothelial cells. This coupling effect may be mediated in CD31-endothelial cells via HIF-1α signaling. Xu et al. [28] found that bone formation and CD31hiEmcnhi endothelial cells increased significantly in osteoblast-specific SHN3-deficient mice. Transcriptomic analysis revealed that SLIT3 is a proangiogenic factor derived from osteoblasts and regulated by shn3. Slit3 gene deletion reduced bone CD31hiEmcnhi endothelial cells, resulting in decreased bone mass due to impaired bone formation, and partially reversed the high bone mass phenotype in Shn3 mice. Defect in fracture repair in slit3 mutant mice and enhanced fracture repair in shn3 mutant mice. All of the above studies suggest that this coupling between osteoblasts and CD31hiEmcnhi endothelial cells is essential for bone healing and that blood vessels are involved in targeting bone anabolism. Angiogene-osteogenesis coupling plays a key role in the process of bone growth, development, remodeling and repair. Therefore, in the study of the mechanism of hormone-induced femoral head necrosis, it is very necessary to consider the influence of glucocorticoids on the angiogenesis and osteogenesis of femoral head necrosis, so as to explore the mechanism of the occurrence and development of femoral head necrosis.

## Head of femur

### Microstructure of femoral head

The femoral head has a special shape and function. It forms a stable and flexible joint with the acetabulum, glenoid, pubic ligament, iliofemoral ligament and ischial ligament, which plays an important role in maintaining the upright posture of the human body and ensuring the smooth completion of various movements. Under electron microscope, the bone columns and plates in the cancellous bone of the femoral head were interwoven into a network to form porous bone tissue. The pillars are 80–150 microns in diameter and are arched and arranged in all directions, and there is a certain gap between each two arched pillars. Trabeculae arched bones have important mechanical effects [29]. In the study of the microstructure of necrotic femoral head,

Inoue [30] collected 76 hip joints from 50 patients diagnosed with ONFH between 2017 and 2021, Groups 1, 2, 3, and 4 included hip joint without ONFH, femur head collapse without FHC, ONFH with mild collapse (< 2 mm), and severe collapse (> 2 mm), respectively. MDCT was performed in all patients to evaluate the microstructure of subchondral trabecular bone. The results showed that in the femoral head and acetabular region, there were significant differences between the necrotic but not collapsed group and the mild collapsed group compared to the normal femoral head, with increased volumetric bone density and apparent bone volume fraction, and more lamelike and increased connectivity, indicating that osteosclerosis changes were occurring. In the study, Pascart [31] found that compared with the femoral head with osteoarthritis, the trabecular volume fraction of the femoral head with osteonecrosis decreased significantly in the necrotic area, but the trabecular thickness was greater in the marginal sclerotic area, which was due to bone reconstruction and repair in the marginal sclerotic area. Baba [32] used micro-computed tomography (micro-CT) to quantize the volume of bone resorption lesions in osteonecrosis (ONFH) after femoral head collapse in 35 patients with different stages of ONFH, and found that the bone resorption volume of ONFH after femoral head collapse was significantly correlated with the disease stage, and the front of the femoral head was more extensive than the back. Gao [33] reconstructed a three-dimensional geometric model of the hip joint by collecting the CT imaging data of the hip joint of volunteers. Based on the Abaqus finite element software, the collapse risk of the necrotic area was evaluated. The results showed that the closer the area was to the load-bearing line, the higher the risk of necrotic collapse. The above studies show that the femoral head has a unique microstructure due to its special anatomical location and physiological effects. However, the microstructure of necrotic femoral head varies in different stages and regions. Generally speaking, with the progression of necrosis, the volume fraction and thickness of bone trabecula in the necrotic area will decrease, resulting in discontinuity and even necrotic collapse. The closer to the load-bearing line, the more significant the necrosis, the more obvious the microstructure loss and the more serious the collapse.

### Blood flow and microvascular structure of femoral head

At present, enhanced computed tomography (CT) scan, ultra-selective digital subtraction angiography (DSA) angiography and enhanced magnetic resonance imaging (MRI) scan are the main methods for clinical research on intraosseous vessels. However, enhanced CT scan, ultra-selective DSA angiography and enhanced MRI scan are limited by the resolution range. The epiphyseal network or the fine blood vessels within the femoral head cannot be shown in pathological conditions such as femoral neck fracture, hip dysplasia or necrosis. With the advent of 3D high resolution imaging, people have discovered the vascular anatomy system that connects the bone vasculature and the bone marrow [34]. This newly discovered vascular system consists of small arteries, veins, and capillaries, collectively known as transcortical vessels (TCV), and reveals a link between endoosseous and periosteal circulation [35].

The femur is a specially structured, highly vascularized bone that is the longest bone in the human body. Its mechanical strength, recovery, repair, regeneration and remodeling depend on vascular health, which contributes to a continuous supply of blood and provides adequate oxygen, nutrients, growth factors and bone progenitor cells to the bones [36]. Therefore, angiogenesis is expected to lead to revascularization, reperfusion, and absorption of the necrotic area. The nutrient artery, a branch of the circulatory system, is the largest blood vessel entering the pulp cavity and supplies almost half of its blood volume to the femur [37]. At the proximal end, it forms an anastomosis with the perforated artery, and at the distal end, it merges with the deep femoral artery. It extends lengthwise to the bone and is divided into lateral femoral circumflex arteries and medial femoral circumflex arteries [38]. The two branches of the lateral circumflex femoral artery supply the femoral head region via the lateral epiphyseal artery and the neck region via the posterior superior supporting band artery [39]. The femoral head ligament is also supplied by the anterior branch of the obturator artery of the hip bone, which passes through the lower part of the pubic branch and anastomosed with the femoral artery and the medial femoral circumflex branch [40]. Qiu [41] dissected and exposed the arteries of femoral head specimens of 12 cases after fracture replacement and performed arterial microperfusion. Micro CT scanning was performed on all femoral heads and a digital 3D model was reconstructed to quantize the internal femoral head arteries. The results showed that all 12 cases had epiphysis arterial network structure and its small vascular branches, indicating that there were also rich and unique microvascular structures in the femoral head.

## Type-H vessels

### Vascular histological characteristics of Type-H

Kusumbe et al. [21] identified a new blood vessel subtype in trabecular and cortical bone near the tibial growth plate and on the periostium and endomucin surfaces of mice, respectively, called Type-H blood vessels due to the specific expression of the antibodies CD31 and endomucin on the surface of their endothelial cells.This vessel is present in the endobone and metaphysis and is filled with mesenchymal cells and bone progenitor cells that mediate subchondral remodeling by coupling angiogenesis and osteogenesis [42]. In contrast, sinusoidal vessels in the backbone form a dense, highly branched network of capillaries in the bone marrow cavity of the backbone with low expression of CD31 and Emcn, called L-type vessels, oxygen-rich blood flows from the arteries and distal arterioles and is directly connected to H-type vessels, then the blood continues to flow to the L-type sinusoidal network at the junction of the metaphyseal and diaphyseal ends, and eventually to the central vein [43]. Gao et al. [44] isolated and cultured H subtype blood vessels from the femoral head of patients undergoing total hip arthroplasty, and proved the existence of H subtype blood vessels in the femoral head of patients, once again proving the existence of microvascular structure in the femoral head. H-type blood vessels have a dense array of bone progenitor cells, which mainly express Osterix and can be differentiated into osteoblasts and osteoblasts. According to Kusumbe et al. [21], Runx2 early bone progenitors and 1α collagen osteoblasts are also densely arranged around CD31 vessels in metaphysis and endobone, that is, H-type vessels. Bone progenitor cells and osteoblasts were absent near L-type vessels in the diaphysial sinus.The close proximity of bone progenitor cells to H-type vessels provided osteoblast resources for bone formation, while the high oxygen content and related factors secreted by H-type vessels provided the necessary nutrients to meet the metabolic needs of bone formation, forming a complete vaso-osteogenic coupling.

### Mechanism of Type-H vessels in angiogenic osteogenic coupling

H-type angiogenesis is closely bound to osteogenesis, which indicates that there is molecular communication between endothelial cells and osteoblasts. Existing studies have identified several factors that regulate H-type angiogenesis and osteogenesis involved in the coupling of angiogenesis and osteogenesis [43]. Osteoclasts, osteoblasts, chondrocytes, and endothelial lineage cells secrete factors that induce vascular endothelial cell (EC) proliferation, vascular assembly, and stability, such as PDGF-BB and SLIT3; ECs can secrete factors that promote vascular assembly and stability, and can also promote bone formation, such as HIF-1α, Notch, and VEGF, and there may be other factors that remain to be clarified.

Platelet-derived growth factor BB type (PDGF-BB) is a chemotactic and mitogenic factor in the PDGF family that is critical for promoting migration, proliferation, and differentiation of various mesenchymal cell types. PDGF-BB secreted by proosteoclasts, immature precursors of absorbent osteoclasts, promotes proliferation, metastasis, and differentiation of endothelial progenitor cells and mesenchymal stem cells to promote angiogenesis and osteogenesis [45].

SLIT guided ligand 3 (SLIT3) is one of three Slit ligands originally identified in the central nervous system, and multiple studies have shown that SLIT3 is expressed in other tissues outside the nervous system and is involved in other physiological functions.In bone, recent studies have shown that both SLIT3 secreted by osteoblasts [28] and SLIT3 secreted by osteoclasts [46] promote H-type blood vessel formation and bone formation, but the primary source of SLIT3 remains controversial.

Hypoxia-inducing factor HIF is a transcription factor that mediates cell activity in response to changes in oxygen and controls physiological and pathological neovascularization [47].The HIF heterodimer consists of one of three α subunits (HIF-1α, HIF-2α, and HIF-3α) and one β subunit. HIF-1α expression and activity are regulated by hypoxia, and target genes are activated and function through one of two mechanisms [48]. HIF-1α target gene may act directly on bone cells, or it may stimulate VEGF production, promote H-type blood vessel formation and invasion of bone. More and more blood vessels provide osteoblastic clues and may provide osteoblastic progenitor cells that then mature and form new bone [49].

Notch signaling pathway affects the generation and osteogenic coupling of H-type blood vessels: Signals transmitted between adjacent cells through Notch receptors can regulate cell differentiation, proliferation and apoptosis. Notch signal transduction is affected by blood flow, while the small diameter of H-type blood vessels leads to high flow rate, which stimulates signal transduction [50]. In the study, Ramasamy [51] increased Notch signaling through genetic manipulation of Notch receptor inactivators (Fbwx) resulted in increased secretion of H-type blood vessels, Runx2 bone progenitor cells, and EC Noggin; In contrast, EC-specific inactivation of Notch mutants by reducing the essential mediator of Notch signaling (RBPJκ) or deltlike 4 in bone after birth resulted in decreased H-type blood vessels, Noggin expression, and EC proliferation. These results suggest that Notch signaling pathway is a key component of molecular crosstalk that connects angiogenesis, vascular secretion and osteogenesis.

Vascular endothelial growth factor (VEGFA): VEGFA is considered a major regulator of angiogenesis and has been extensively studied in bone [26]. VEGFA promotes the migration and diffusion of endothelial cells (EC). Chondrocytes, osteoclasts, and osteoblasts secrete VEGFA, and the transcription and secretion of VEGFA can be induced by a variety of cytokines and transcription factors, such as HIF-1α, PDGF-BB, and Runx2 [52].

## Effects of glucocorticoids on microvessels of femoral head

Glucocorticoid-induced ischemic necrosis of the femoral head is mainly caused by three important pathways, namely, coagulation dysfunction, endothelial dysfunction and impaired angiogenesis [53].

### Coagulation disorders

Coagulation dysfunction refers to the disturbance of homeostasis of pro-coagulation factors and anticoagulation factors, excessive thrombosis, and the reduction of thrombolysis [54]. The coagulation dysfunction of femoral head necrosis is affected by two molecular pathways, namely, thrombolysis and hypofibrinolysis. Hypercoagulability, also known as hypercoagulability, is an abnormality of the blood clotting mechanism that promotes the formation of blood clots in the walls of circulating blood vessels [55], characterized by an increase in pro-coagulant factors or a decrease in natural anticoagulants [56]. Glucocorticoid-induced fibrinolysis is the process of breaking down blood clots or clots that is tightly regulated by activators such as tissue plasminogen activators (TPA) and urokinase-type plasminogen activators (UPA), as well as by inhibitors such as tissue factor plasminogen inhibitors (TFPI) and plasminogen activator inhibitor-1(PAI-1), as well as plasminogen proteinases. Glucocorticoids decreased fibrinolytic protein activity and relative hypercoagulability by increasing PAI-1 level and decreasing TPA level [23, 57]. In the study, Okada et al. [58] used female wild-type mice with PAI-1 gene deficiency to give 2 mg/kg/d dexamethasone (Dex) intraperitoneally for 3 weeks, found that dexamethasone (Dex) increased plasma PAI-1 levels and PAI-1 mRNA levels in adipose-tissue and muscle of wild-type mice, while Pai-1 deficiency significantly attenuated the slow bone repair caused by Dex, suggesting that increased PAI levels cause slower bone repair. Lu Li et al. [59] detected significantly increased expression of PAI-1 in the local microenvironment of the femoral head in a steroid-induced rabbit ONFH model (MPS treatment, 20 mg/kg/d, lasting for 3 d).All the above studies have shown that glucocorticoids can cause significant expression of PAI-1 in the local microenvironment of the femoral head, inhibit plasminogen from dissolving thrombus or clot, lead to microvascular hypercoagulation of the femoral head, coagulation dysfunction, damage blood flow of the femoral head, and lead to necrosis of the femoral head.

### Endothelial cell dysfunction

Vascular endothelial cells form the inner layer of blood vessels, they play a key role in the development and maintenance of the functional circulatory system and provide paracrine support to surrounding non-vascular cells [60]. Under various physical and chemical stimulation, the endocrine, paracrine and autocrine functions are activated to produce vasodilators and vasodilators [61]. Endothelial cells regulate homeostasis by maintaining the balance between vasodilators and vasoconstrictors, anticoagulants and coagulants, inflammatory and anti-inflammatory molecules, oxidants and antioxidants, and fibrinolytic agents and antifibrinolytic agents. Loss of homeostasis is called endothelial dysfunction, which can lead to a series of circulatory diseases, such as thrombosis and coagulation disorders [62]. Bone microvascular endothelial cells (BMECs) are highly active endocrine cells, which are composed of single-layer structures attached to the inner wall of bone to form bone microvessels [63]. The injury of glucocorticoid to BMEC can lead to local blood hypercoagulation, microvascular thrombosis and vascular occlusion, which is one of the main reasons for the dysfunction of femoral head endothelial cells. By studying femoral head tissues of patients with hormone-induced femoral head necrosis, Huang et al. [64] found that glucocorticoid stimulation significantly inhibited cell viability, promoted apoptosis and increased mRNA expression of pro-inflammatory cytokines (such as TNF-α, IL-1β and IFN-γ) in human umbilical vein endothelial cells (HUVECs) and BMEC. By analyzing a comprehensive dataset of gene expression to identify dysregulated mirnas in ONFH, it was found that miR-122-5p was underexpressed in GC-induced ONFH femoral head tissue and GC-stimulated bone microvascular endothelial cells (BMEC), while overexpressed miR-122-5p significantly inhibited GC-induced endothelial cell damage. Yu et al. [65] isolated BMECs from the subchondral region of the femoral head in patients with hormone-induced femoral head necrosis and in patients with femoral neck fracture. Cell proliferation, cell viability, tube formation test, Transwell test, TUNEL assay and Western blot analysis were performed. The results showed that the cell viability, angiopoiesis and migration of BMECs in the glucocorticoid-induced ONFH group significantly decreased, and the number of TUNEL-positive cells significantly increased. These results suggest that glucocorticoids can lead to decreased angiogenesis and increased apoptotic activity of BMECs in femoral head.In summary, GC can inhibit the activity of endothelial cells, promote their apoptosis, increase the expression of proinflammatory cytokines mRNA, cause dysfunction of femoral head microvascular endothelial cells, and promote the process of femoral head necrosis.

### Impaired angiogenesis

Angiogenesis is the process of developing new blood vessels from existing blood vessels, which are considered the most important organs in embryonic development [66]. During ischemia, endothelial cells receive stimulation from the local environment and initiate the angiogenic program, a dynamic process that utilizes a balance between pro-angiogenic and anti-angiogenic factors, resulting in the expansion of the vascular network [67]. Vascular and bone-derived angiogenic factors and stimuli play a role in angiogenesis, such as Hypoxia-inducible fact-1α (HIF-1α) and vascular endothelial growth factor (VEGF), vascular endothelial growth factor receptors (VEGFR), Vascular endothelial growth factor receptors (VEGF), Vascular endothelial cadherin (VEGFR) VE-cadherin, cluster of differentiation31, CD31), delta-like typical Notch ligand 4-Notch-Noggin (DLL4-NOTCH-NOG), etc. The molecular pathways between the vasculature and bone interact and work synergistically to initiate the angiogene-osteogenic coupling to promote overall regenerative repair of necrotic areas [68]. When cells after ischemia, the tissue hypoxia state, cells in low factor (alpha hypoxia inducible factor 1 alpha, HIF -1α) will be a large increase, increase vascular endothelial growth factor (vascular endothelial growth factor, VEGF) expression level, Thereby promoting the repair and regeneration of blood vessels [69]. However, glucocorticoids disrupt this repair mechanism. Xu et al. [70] found that when MC3T3-E1 cells were exposed to different concentrations of dexamethasone (Dex), the expression of HIF-1α protein was decreased, and the expression of M.PK1 in MC3T3-E1 cells was also decreased after the concentration range of 10^–9^–10^–6^ dexamethasone treatment. When treated with glucocorticoid receptor antagonists RU486 and dexamethasone, MC3T3-E1 cells showed enhanced HIF-1α expression, indicating that glucocorticoids can reduce the expression of HIF-1α. This study also showed that the up-regulated expression of HIF-1α can promote the osteogenic ability of MC3T3-E1 cells and the expression of PDK1. Liu et al. [71] demonstrated that glucocorticoid therapy inhibits angiogenin (ANG) production by inhibiting osteoclast formation in metaphyseal region, leading to impaired endothelial rRNA transcription and subsequent cell senescence. Glucocorticoids can also damage microvascular endothelial cells in the bone of the femoral head, causing endothelial cell dysfunction, resulting in reduced angiogenesis of BMECs [40, 41]. Therefore, glucocorticoids can inhibit angiogenesis, destroy osteoblastation-angiogenesis coupling, and cause femoral head necrosis by decreasing HIF-1α protein expression, inhibiting ANG production and BMECS activity.

In summary, glucocorticoids can cause coagulation dysfunction, endothelial dysfunction and angiogenesis disorders, leading to the destruction of microvascular blood flow in the femoral head, resulting in cell ischemia and hypoxia, and affecting bone metabolism. Ischemia and hypoxia can reduce the osteogenic differentiation of peripheral blood mesenchymal stem cells by up-regulating Notch-1 expression [72], further reduce osteogenesis, destroy the osteoblastation-angiogenesis coupling, and eventually lead to the occurrence of femoral head necrosis.

## Effect of glucocorticoid on Type-H vessels in femoral head

Type-H vessels are the key microvessels in the femoral head, which play an important role in maintaining blood flow in the femoral head and osteogenic angiogenic coupling [25–27], the femoral head is the anatomic site sensitive to the adverse effects of glucocorticoids [73]. Long-term use of glucocorticoids will affect the expression of factors related to the regulation of H-type blood vessels, thereby causing serious damage to H-type blood vessels.

### Glucocorticoids decreased HIF-1α and VEGF expression

H-type vessels are regulated by hypoxia-inducible factor (HIF-1α) and vascular endothelial cell growth factor (VEGF). HIF-1α is a transcription factor that mediates cell activity in response to changes in oxygen and controls physiological and pathological neovascularization [48]. VEGF is a major regulator of angiogenesis and promotes migration and diffusion of endothelial cells (EC) [26]. The stability and activity of the HIF-1α subunit is regulated by its post-translational modifications such as hydroxylation, ubiquitination, acetylation, and phosphorylation [74]. Under normal oxygen conditions, PHD hydroxylated proline (Pro) residues are present in ODDD (Pro 402 and 564 of HIF1α, Pro 405 and 531) of HIF2α induce ubiquitin reaction of E3 ubiquitin ligase Von Hippel-Lindau protein (pVHL), mediated by 26S proteasome, resulting in HIF-1α degradation via the ubiquitin proteasome pathway [75]. During hypoxia or iron deficiency, PHDs are inactivated, HIF-1α hydroxylation is inhibited, HIF-1α subunits become stable and dimerize with HIF-1β, forming an HIF complex via ARnt-mediated transfer from cytoplasmic to nuclear, possibly interacting with HIF-αβ transcription complex to further activate hypoxia. HIF-αβ heterodimer complexes bind to hypoxia response elements (HREs) containing the consensus core sequence RCGTG (R: A/G) of target gene promoter region and p300/CBP to regulate the expression of target genes [76]. However, in steroid-induced necrosis of the femoral head, the main target gene of HIF-1α is VEGF [77]. In animal experiments related to glucocorticoids and H-type blood vessels, Laneet al.’s [78] study showed that the number of H-type blood vessels in the distal metaphyseal of femur of mice in the glucocorticoid-treated group (fed 4 mg/L/d Dex, the duration was 4 weeks and the first, 14th and 28th days were observed) was significantly reduced compared with the normal control group and the treatment group. In the femoral head, osteoclasts increased, osteoblasts decreased, HIF-1α and VEGF expression decreased, and the decrease of HIF-1α expression further resulted in the decrease of the number of H subtype vessels. Weinstein et al. [73] used methylprednisolone(MPS) (Full implantable drug delivery system release 2.1 mg/kg/d) to intervene mice to observe the molecular, biomechanical, cellular and vascular changes of femur, and found that after 14 days of intervention, the expression of hypoxia-inducing factor (HIF-1α) and vascular endothelial growth factor (VEGF) in the femoral head, the number of osteoblasts, the rate of bone formation and the strength of bone decreased, while osteoclasts increased. In addition, the decrease of Hif-1α and VEGF expression, bone vessels and strength preceded the loss of bone mass and the deterioration of microstructure, which made the femoral head prone to collapse. Yu al. [79] used MPS combined with LPS to perform SINFH modeling. Rats were injected intraperitoneally with lipopolysaccharides (LPS 20 μg/kg) for two consecutive times at an interval of 24h each time. After the last injection 24h later, meprednisolone (40 mg/kg) was alternately injected into both gluteus muscles for three times, at an interval of 24 h each time. In the desferramine group, intraperitoneal injection of desferramine mesylate (250 mg/kg) was administered. After 6 weeks, Micro-CT analysis was used to observe the changes in the microstructure of the femoral head, HE staining was used to observe the pathological changes of the femoral head, and immunofluorescence staining was used to analyze the changes in H-type blood vessels in the femoral head. RT-PCR was used to analyze the expression of Hif-1α/VEGF signaling axis related factors in the femoral head. The results showed that the expressions of Hif-1α and VEGF were significantly lower than those in the injection group, and H-type angiogenesis was reduced, bone trabeculae were thin, and microfractures occurred. This was because the degradation of HIF-1α could be inhibited by the reduction of intracellular Fe2^+^ concentration and the inhibition of PHD activity.

**Fig. 1:**
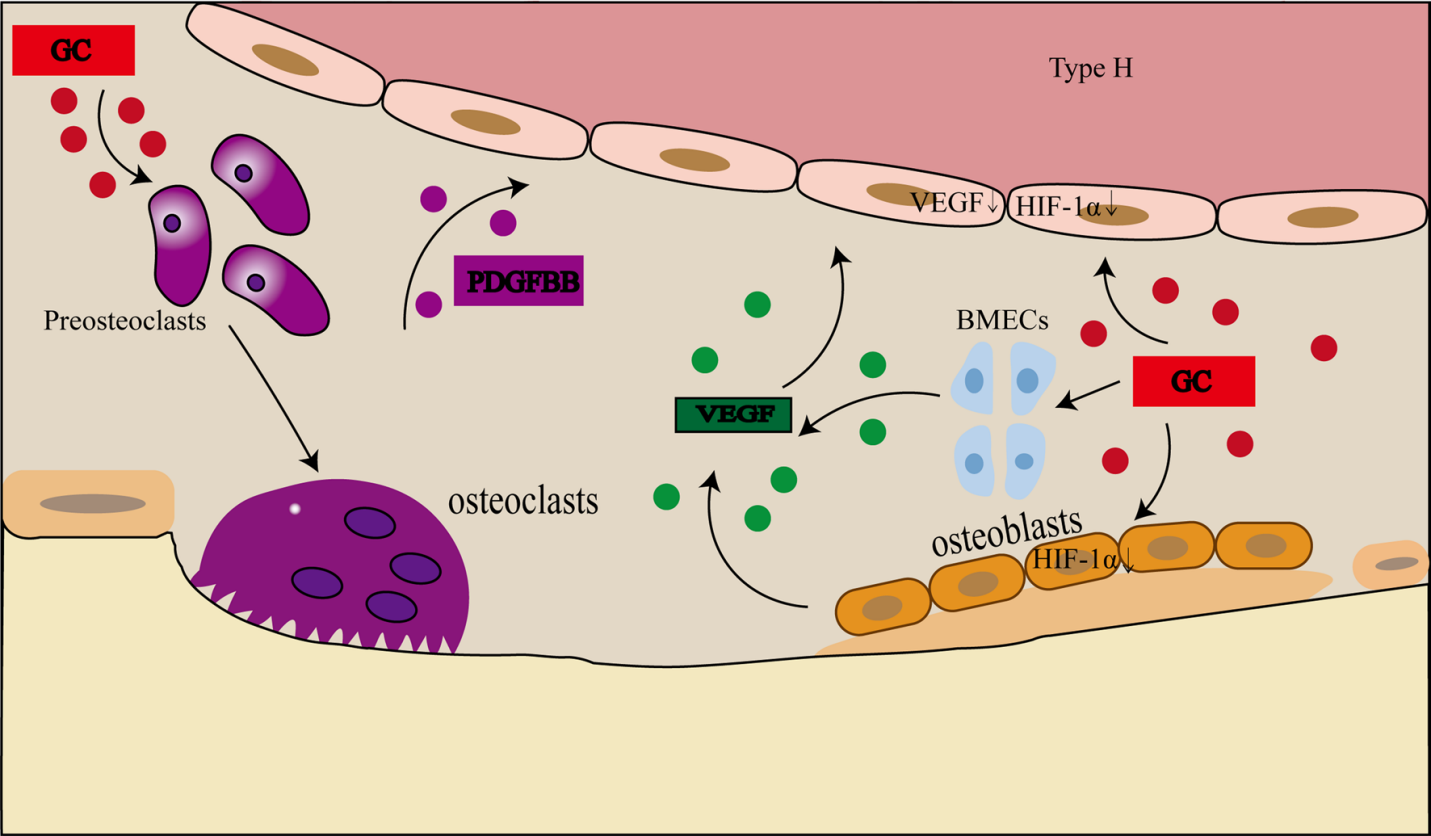
Glucocorticoids inhibited the expression of HIF-1α, VEGF and PDGF-BB

According to the above studies, combined with the action mechanism of HIF-1α and VEGF, it can be seen that glucocorticoids reduce the expression of HIF-1α in the femoral head of mice, thereby reducing the expression of VEGF, leading to the decrease of H-type angiogenesis, and the lack of vascular endothelial cells due to the inhibition of H-type angiogenesis will further cause the decrease of HIF-1α expression (but this is not the main reason for the decrease of HIF-1α), and then disruption of bone microcirculation, and influence bone metabolism (Fig. 1).

### Glucocorticoids inhibited PDGF-BB expression

H-type vessels are regulated by pre-osteoclast (POC) platelet-derived growth factor BB (PDGF-BB) and bind specifically to bone formation and development. Li et al. [80] established the glucocorticoid-induced osteoporosis group (GIOP group) by intraperitoneal injection of MPS 10 mg/m^2^/d for four weeks. PDGF-BB levels in bone marrow and serum were measured. Micro-CT scanning was performed on the femur, frozen sections of the femur were taken, and CD31hiEmcnhiH type vascular immunofluorescence staining was performed. The results showed that bone mass decreased in GIOP group compared with control group. The expression of PDGF-BB in serum and bone marrow of GIOP group was significantly decreased, and the expressions of CD31hi and Endomucinhi were downregulated. The results showed that long-term use of glucocorticoids can inhibit the secretion of PDGF-BB in osteoclast precursor cells of mice, thus inhibiting the growth of H-type blood vessels specific for bone growth. Peng et al. [81] conducted MPS (10mg/m^2^/d for 2, 4, 6weeks) intervention in two weeks old mices to model hormone-induced femoral head necrosis. After 2 weeks, it was found that POCs in the femoral head remained unchanged and POC synthesis of PDGF-BB decreased. The longer the duration of MPS treatment, the fewer POCs and PDGF-BB, H-type blood vessels, bone formation rate, bone mass and bone length. Shangguan et al. [82] used hypodermic injection of dexamethasone (0.2 mg/kg/d) in rats at days 9–20 and found that dexamethasone promoted the expression of glucocorticoid receptor (GR), CCAAT and enhancer binding protein α (C/EBP-α) and miR-34c in female fetal bone. In addition, the expression of bone mass, H-type angiogenesis and genes related to the bone platelet-derived growth factor receptor β (PDGFRβ)/adhesion plaque kinase (FAK) pathway was decreased in prenatal and postnatal female offspring, suggesting that glucocorticoids also inhibit the bone platelet-derived growth factor receptor β (PDGFRβ)/adhesion plaque kinase (FAK) pathway. Inhibit the formation of H-type blood vessels, thereby affecting bone metabolism and osteo-vascular coupling, resulting in femoral head necrosis. Yang et al. [83] reported that glucocorticoids inhibited the secretion of PDGF-BB before osteoclasts, resulting in the inhibition of H-type blood vessels and reduced osteogenesis. In young GIO mouse models, mice were intraperitoneally injected with methylprednisolone (MPS) at 10mg/m^2^/day from 2 to 6 weeks of age, and the cathepsin K inhibitor L-235 was found to prevent bone loss by inhibiting osteoclast bone resorption while maintaining preosteoclast secretion of PDGF-BB and preserving H-type blood vessels. Above studies have shown that glucocorticoids inhibit the synthesis of PDGF-BB in POCs (Fig. 1), thereby inhibiting the formation of H-type blood vessels, resulting in the destruction of blood transport and osteogenesis-angiogenesis coupling of femoral head, reduced osteogenesis, trabecular fracture, and even necrotic collapse.

### Glucocorticoid inhibits ANG production

**Fig. 2:**
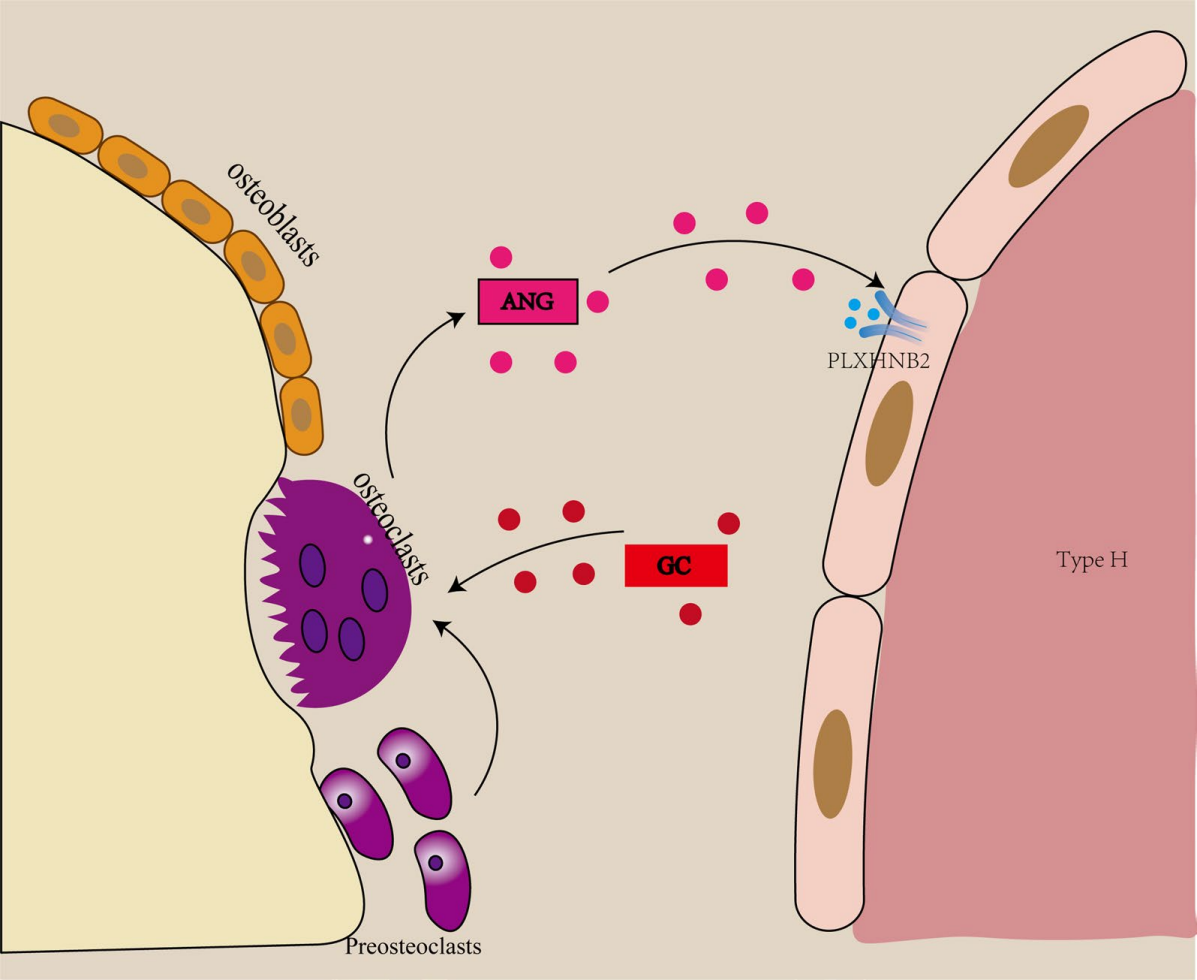
Glucocorticoids inhibit ANG production by osteoclasts and thus inhibit H-type angiogenesis

Angiogenin (ANG) is a secretory ribonuclease with growth-promoting activity [84], which mainly stimulates the growth and proliferation of endothelial cells by promoting the transcription of 47S rRNA and affects angiogenesis [85]. In recent years, plexin-B2 (PLXNB2) has been found to be a transmembrane receptor belonging to the plexin family and has been identified as a functional ANG receptor [86, 87]. ANG maintains the proliferative activity of endothelial cells through ribosomal RNA (rRNA) transcription mediated by plexin-B2 (PLXNB2). GC treatment inhibits ANG production by inhibiting osteoclast formation in metaphyseal, leading to impaired endothelial rRNA transcription and subsequent cell senescence. Liu et al. [71] detected by immunofluorescence staining that MPS treatment (10 mg/m^2^/d, intraperitoneal injection,for two weeks) induced a time-dependent reduction of CD31hiEmcnhi blood vessels in primary and secondary ONFH of little mouse.. Flow cytometry analysis showed that compared with carrier treated mice, the number of CD31hiEmcnhi and CD31loEmcnlo cells in the same region of mice treated by MPS decreased, and the CD31hiEmcnhi type H blood vessels were more profoundly affected. This study showed that osteogenic coupled H blood vessels are the main targets of GCs, and GCs can inhibit osteoclast formation and ANG production. Inhibit the formation of H-type vessels (Fig. 2).

## Discussion

There is complex crosstalk between GC and HIF-1α: Vettori et al. [88] using zebrafish in vivo studies showing that GC plays a role in promoting C-SrC-mediated pVHL proteomic degradation, and that GC stabilizes the HIF model by activating c-src and subsequent pVHL instability, thereby effectively activating HIF-1α transcriptional responses. However, Wagner et al. [89] showed that HIF-1α activity was weakened under hypoxia and DEX stimulation due to decreased DNA binding and HRE activity associated with HIF-1α nuclear translocation problems, revealing the related inhibitory effect of dexamethasone on HIF-1α target gene expression in hypoxic HEPG2 cells. In particular, by western blotting analysis, they showed that dexamethasone reduced nuclear HIF-1α protein because the amount of HIF-1α in the cytoplasmic extract was higher than the amount of HIF-1α in the nuclear extract after DEX treatment. This cytoplasmic retention of HIF-1α suggests that nuclear input is blocked through a still unknown mechanism, leading to reduced expression of HIF target genes. In addition, by using luciferase assays, the authors found that dexamethasone not only weakened HIF-1 activity in a GR-dependent manner, but that this effect depended on the presence of functional HRE. Over the next few years, Gaberet al. [90] investigated the interaction between the roles of macrophage migration inhibitors (MIF), HIF, and GCs in human primary non-tumor CD4 + Th cells and Jurkat T cells. Compared to previous observations, this study demonstrated the presence of a significant dose-dependent dexamethasone inhibition of HIF-1α protein expression, resulting in reduced HIF-1 target gene expression. Thus, as an alternative to Wagner et al.’s 2008 hypothesis, they proposed a model based on either fast DeX-mediated induction of HIF-1α inhibitors (e.g. PHD1-3, FIH, and pVHL) or fast DeX-mediated hypoxia-induced signaling inhibition. These different in vitro data reflect the diversity that exists between the cell types used in these studies. In the ONFH study, the effect of GC on HIF-1α was consistent with the results of Wagner, Gaber et al.

It is worth noting that GC leads to decreased expression of HIF-1α promoting angiogenesis factor, resulting in inhibition of angiogenesis, which means insufficient H-type vascular endothelial cells and further leads to decreased expression of HIF-1α. However, HIF-1α is not only found in vascular endothelial cells in the femoral head. HIF-1α is present in osteocytes, osteoblasts, osteoclasts, preosteoclasts and endothelial progenitor cells, so the decrease of HIF-1α caused by angiogenesis inhibition is not the main factor. In addition to HIF-1α, other factors such as PDGF-BB are also involved in angiogenesis inhibition caused by glucocorticoid action, and the reduction of HIF-1α is not the only determinant.

The mechanism of necrosis of the femoral head is complex and diverse. Currently, the pathogenesis of ONFH includes the theory of intravascular coagulation, the theory of fat metabolism disorder, the theory of intraosseous hypertension, and the theory of arteriovasculitis, etc [88, 91]. However, there are few studies on the changes of blood flow microstructure of bone tissue. Based on the microvascular structure of the femoral head and its effect on the osteogenesis and trabecular structure of the femoral head, this review summarized and analyzed the microvascular injury of the femoral head caused by glucocorticoids, providing a new idea for the study of the mechanism of femoral head necrosis from the perspective of osteogenesis and vascular coupling. Most of the existing studies on the pathogenesis are based on animal modeling, and there is a lack of studies on femoral head specimens. However, specimens of normal and different types of necrosis and different stages of necrosis are easy to obtain. The mechanism of necrosis of the femoral head is mainly ischemia, but the related mechanism of necrosis tissue ischemia has not been explored, especially in the microcirculation of the femoral head, bone trabecula and other microscopic structures. The femoral head has numerous microvessels, and H-type vessels are the most critical in promoting osteogenesis and inhibiting osteoclastic bone remodeling. There is a lack of research on the changes of H-type vessels and their mechanisms during femoral head necrosis.

## Conclusion

When femoral head necrosis occurs, there is a complex injury and repair process. However, due to the destruction of the balance between bone resorption and bone formation, the injury is greater than the repair, resulting in necrosis and even collapse. Bone reconstruction and repair are inseparable from the vaso-osteogenesis coupling. H subtype vessels are a newly discovered intraosseous vessels, mainly distributed in metaphyseal and endoosseum. They are highly expressing endothelial mucin and CD31 and secreting growth factors related to the survival and proliferation of bone progenitor cells. A large number of Osterix positive bone progenitor cells, type I α collagen positive osteoblasts and Runx2 positive early bone progenitor cells were gathered around, which could secrete VEGF to promote angiogenesis and have the ability to induce bone formation and angiogenesis. PDGF-BB, fissure guiding ligand 3, HIF-1α, Notch signaling pathway and VEGF are involved in the biological mechanism of H subtype blood vessels promoting osteogenesis. There are abundant microvessels in the femoral head, and H-type vessels play a key role in bone reconstruction and repair. During the occurrence of hormone-induced femoral head necrosis, the vaso-osteogenic coupling is severely damaged. The mechanism may be that glucocorticoids inhibit the formation of H-type blood vessels by decreasing the expression of HIF-1α and VGEF, leading to the disturbance of NONFH microcirculation, thus causing damage to the vaso-bone remodeling coupling. It reduces the ability of reconstruction and repair during the occurrence and development of femoral head necrosis and promotes the progression of hormone-induced femoral head necrosis.

## References

[1] Choi H-R, Steinberg ME, Cheng EY (2015). Osteonecrosis of the femoral head: diagnosis and classification systems. Curr Rev Musculoskelet Med.

[2] Singh S, Yadav SK, Meena VK (2023). Orthopedic scaffolds: evaluation of structural strength and permeability of fluid flow via an open cell neovius structure for bone tissue engineering. ACS Biomater Sci Eng.

[3] Jianxiong M, Weiwei H, Jie Z (2017). Recent progress in pathogenesis of femoral head necrosis. J Tissue Eng.

[4] Cohen-Rosenblum A, Cui Q (2019). Osteonecrosis of the femoral head. Orthop Clin North Am.

[5] Lin Q, Ren Z. Research progress on the mechanism of action of Chinese medicine in the treatment of femoral head necrosis. Chin J Pharmacoeconomics;17:121–4 (**in Chinese**).

[6] Petek D, Hannouche D, Suva D (2019). Osteonecrosis of the femoral head: pathophysiology and current concepts of treatment. EFORT Open Rev.

[7] Mont MA, Salem HS, Piuzzi NS (2020). Nontraumatic osteonecrosis of the femoral head. J Bone Jt Surg.

[8] Zhao D, Zhang F, Wang B (2020). Guidelines for clinical diagnosis and treatment of osteonecrosis of the femoral head in adults. J Orthop Transl.

[9] Sadile F, Bernasconi A, Russo S (2016). Core decompression versus other joint preserving treatments for osteonecrosis of the femoral head: a meta-analysis. Br Med Bull.

[10] Wu CT, Shih-Hsiang Y, Lin PC (2018). Long-term outcomes of Phemister bone grafting for patients with non-traumatic osteonecrosis of the femoral head. Int Orthop.

[11] Chen L, Hong G, Hong Z (2020). Optimizing indications of impacting bone allograft transplantation in osteonecrosis of the femoral head. Bone Jt J.

[12] Wang C, Xie Q, Yang L (2020). A 3D printed porous titanium alloy rod with biogenic lamellar configuration for treatment of the early-stage femoral head osteonecrosis in sheep. J Mech Behav Biomed Mater.

[13] Migliorini F, Maffulli N, Eschweiler J (2021). Core decompression isolated or combined with bone marrow-derived cell therapies for femoral head osteonecrosis. Expert Opin Biol Ther.

[14] Quaranta M, Miranda L, Oliva F (2021). Osteotomies for avascular necrosis of the femoral head. Br Med Bull.

[15] Migliorini F, La Padula G, Oliva F (2022). Operative management of avascular necrosis of the femoral head in skeletally immature patients: a systematic review. Life.

[16] Migliorini F, Maffulli N, Baroncini A (2023). Prognostic factors in the management of osteonecrosis of the femoral head: a systematic review. Surgeon.

[17] Shibin Li, Lai Yu, Yi Z (2019). The pathogenesis of hormone-induced femur head necrosis and the target effect of related signaling pathways. J Tissue Eng.

[18] Weinstein RS (2011). Clinical practice. Glucocorticoid-induced bone disease. N Engl J Med.

[19] Hines JT, Jo WL, Cui Q (2021). Osteonecrosis of the femoral head: an updated review of ARCO on pathogenesis, staging and treatment. J Korean Med Sci.

[20] Timmermans S, Souffriau J, Libert C (2019). A General introduction to glucocorticoid biology. Front Immunol.

[21] Kusumbe AP, Ramasamy SK, Adams RH (2014). Coupling of angiogenesis and osteogenesis by a specific vessel subtype in bone. Nature.

[22] Kim JE (2022). Osteoclastogenesis and osteogenesis. Int J Mol Sci.

[23] Kim KJ, Lee J, Wang W (2021). Austalide K from the fungus *Penicillium rudallense* prevents LPS-induced bone loss in mice by inhibiting osteoclast differentiation and promoting osteoblast differentiation. Int J Mol Sci.

[24] Zheng ZW, Chen YH, Wu DY (2018). Development of an accurate and proactive immunomodulatory strategy to improve bone substitute material-mediated osteogenesis and angiogenesis. Theranostics.

[25] Percival CJ, Richtsmeier JT (2013). Angiogenesis and intramembranous osteogenesis. Dev Dyn.

[26] Grosso A, Burger MG, Lunger A (2017). It takes two to tango: coupling of angiogenesis and osteogenesis for bone regeneration. Front Bioeng Biotechnol.

[27] Wang Q, Zhou J, Wang X (2022). Coupling induction of osteogenesis and type H vessels by pulsed electromagnetic fields in ovariectomy-induced osteoporosis in mice. Bone.

[28] Xu R, Yallowitz A, Qin A (2018). Targeting skeletal endothelium to ameliorate bone loss. Nat Med.

[29] Song W, Tao Z, Xinlong Ma (2015). Research progress on the role of biomechanical factors in the occurrence and development of femoral head necrosis. Shandong Med.

[30] Inoue T, Shoji T, Kato Y (2023). Investigating the subchondral trabecular bone microstructure in patients with osteonecrosis of the femoral head using multi-detector row computed tomography. Mod Rheumatol.

[31] Pascart T, Falgayrac G, Cortet B (2022). Subchondral involvement in osteonecrosis of the femoral head: insight on local composition, microstructure and vascularization. Osteoarthr Cartil.

[32] Baba S, Motomura G, Ikemura S (2020). Quantitative evaluation of bone-resorptive lesion volume in osteonecrosis of the femoral head using micro-computed tomography. Jt Bone Spine.

[33] Gao Y, Fu Y, Xin Z, et al. Prediction of mechanical properties and collapse risk of avascular necrotic femoral head under walking exercise [J/OL]. Chin Tissue Eng Res. 2024.

[34] Grüneboom A, Kling L, Christiansen S (2019). Next-generation imaging of the skeletal system and its blood supply. Nat Rev Rheumatol.

[35] Grüneboom A, Hawwari I, Weidner D (2019). A network of trans-cortical capillaries as mainstay for blood circulation in long bones. Nat Metab.

[36] Shujuan W, Liao M, Xuzhong Z, Yan L. Assessment of local blood supply during femoral neck fracture: advances in anatomical studies and clinical applications. 36(3):294–8 (2023). 10.12200/j.issn.1003-0034.2023.03.020 36946027

[37] Kawasaki Y, Kinose S, Kato K (2020). Anatomic characterization of the femoral nutrient artery: application to fracture and surgery of the femur. Clin Anat.

[38] Rajani SJ, Ravat MK, Rajani JK (2015). Cadaveric study of profunda femoris artery with some unique variations. J Clin Diagn Res.

[39] Zhao D, Qiu X, Wang B (2017). Epiphyseal arterial network and inferior retinacular artery seem critical to femoral head perfusion in adults with femoral neck fractures. Clin Orthop Relat Res.

[40] Lee E-J, Jain M, Alimperti S (2021). Bone microvasculature: stimulus for tissue function and regeneration. Tissue Eng Part B Rev.

[41] Qiu X, Cheng LL, Wang BJ, Liu BY, Yang L, Yu M, Gu G, Zhao DW (2018). Micro perfusion and quantitative analysis of the femoral head intraosseous artery. Orthop Surg.

[42] Xu Z, Kusumbe AP, Cai H (2023). Type H blood vessels in coupling angiogenesis-osteogenesis and its application in bone tissue engineering. J Biomed Mater Res B Appl Biomater.

[43] Peng Y, Wu S, Li Y (2020). Type H blood vessels in bone modeling and remodeling. Theranostics.

[44] Gao F, Mao T, Zhang Q (2020). H subtype vascular endothelial cells in human femoral head: an experimental verification. Ann Palliat Med.

[45] Joshi AA, Padhye AM, Gupta HS (2019). Platelet derived growth factor-BB levels in gingival crevicular fluid of localized intrabony defect sites treated with platelet rich fibrin membrane or collagen membrane containing recombinant human platelet derived growth factor-BB: a randomized clinical and biochemical study. J Periodontol.

[46] Kim BJ, Lee YS, Lee SY (2018). Osteoclast-secreted SLIT3 coordinates bone resorption and formation. J Clin Invest.

[47] Yang Z, Huang Y, Zhu L (2021). SIRT6 promotes angiogenesis and hemorrhage of carotid plaque via regulating HIF-1α and reactive oxygen species. Cell Death Dis.

[48] Negri AL (2022). Role of prolyl hydroxylase/HIF-1 signaling in vascular calcification. Clin Kidney J.

[49] Riddle RC, Khatri R, Schipani E (2009). Role of hypoxia-inducible factor-1alpha in angiogenic-osteogenic coupling. J Mol Med.

[50] Ramasamy SK, Kusumbe AP, Schiller M (2016). Blood flow controls bone vascular function and osteogenesis. Nat Commun.

[51] Ramasamy SK, Kusumbe AP, Wang L (2014). Endothelial Notch activity promotes angiogenesis and osteogenesis in bone. Nature.

[52] Ribatti D, d'Amati A (2023). Bone angiocrine factors. Front Cell Dev Biol.

[53] Singh M, Singh B, Sharma K (2023). A molecular troika of angiogenesis, coagulopathy and endothelial dysfunction in the pathology of avascular necrosis of femoral head: a comprehensive review. Cells.

[54] Palta S, Saroa R, Palta A (2014). Overview of the coagulation system. Indian J Anaesth.

[55] Arachchillage DJ, Mackillop L, Chandratheva A (2022). Thrombophilia testing: a British Society for Haematology guideline. Br J Haematol.

[56] Campello E, Spiezia L, Adamo A (2019). Thrombophilia, risk factors and prevention. Expert Rev Hematol.

[57] Zhang Q, Lv J, Jin L (2018). Role of coagulopathy in glucocorticoid-induced osteonecrosis of the femoral head. J Int Med Res.

[58] Okada K, Okamoto T, Okumoto K (2020). PAI-1 is involved in delayed bone repair induced by glucocorticoids in mice. Bone.

[59] Li L, Wang Y, Yu X (2020). Bone marrow mesenchymal stem cell-derived exosomes promote plasminogen activator inhibitor 1 expression in vascular cells in the local microenvironment during rabbit osteonecrosis of the femoral head. Stem Cell Res Ther.

[60] Trimm E, Red-Horse K (2023). Vascular endothelial cell development and diversity. Nat Rev Cardiol.

[61] Pacinella G, Ciaccio AM, Tuttolomondo A (2022). Endothelial dysfunction and chronic inflammation: the cornerstones of vascular alterations in age-related diseases. Int J Mol Sci.

[62] Wolf D, Ley K (2019). Immunity and inflammation in atherosclerosis. Circ Res.

[63] Ma J, Shen M, Yue D (2022). Extracellular vesicles from BMSCs prevent glucocorticoid-induced BMECs injury by regulating autophagy via the PI3K/Akt/mTOR pathway. Cells.

[64] Huang X, Jie S, Li W, Li H, Ni J, Liu C (2022). miR-122-5p targets GREM2 to protect against glucocorticoid-induced endothelial damage through the BMP signaling pathway. Mol Cell Endocrinol.

[65] Yu H, Liu P, Zuo W (2020). Decreased angiogenic and increased apoptotic activities of bone microvascular endothelial cells in patients with glucocorticoid-induced osteonecrosis of the femoral head. BMC Musculoskelet Disord.

[66] Kretschmer M, Rüdiger D, Zahler S (2021). Mechanical aspects of angiogenesis. Cancers.

[67] Kazerounian S, Lawler J (2018). Integration of pro- and anti-angiogenic signals by endothelial cells. J Cell Commun Signal.

[68] Han Y, You X, Xing W (2018). Paracrine and endocrine actions of bone-the functions of secretory proteins from osteoblasts, osteocytes, and osteoclasts. Bone Res.

[69] Kar S, Samii A, Bertalanffy H (2015). PTEN/PI3K/Akt/VEGF signaling and the cross talk to KRIT1, CCM2, and PDCD10 proteins in cerebral cavernous malformations. Neurosurg Rev.

[70] Xu WN, Zheng HL, Yang RZ (2020). HIF-1α regulates glucocorticoid-induced osteoporosis through PDK1/AKT/mTOR signaling pathway. Front Endocrinol.

[71] Liu X, Chai Y, Liu G (2021). Osteoclasts protect bone blood vessels against senescence through the angiogenin/plexin-B2 axis. Nat Commun.

[72] Yang M, Liu H, Wang Y (2019). Hypoxia reduces the osteogenic differentiation of peripheral blood mesenchymal stem cells by upregulating Notch-1 expression. Connect Tissue Res.

[73] Weinstein RS, Hogan EA, Borrelli MJ (2017). The Pathophysiological sequence of glucocorticoid-induced osteonecrosis of the femoral head in male mice. Endocrinology.

[74] Ke Q, Costa M (2006). Hypoxia-inducible factor-1 (HIF-1). Mol Pharmacol.

[75] Marchi D, van Eeden FJM (2021). Homeostatic regulation of glucocorticoid receptor activity by hypoxia-inducible factor 1: from physiology to clinic. Cells.

[76] Vanderhaeghen T, Beyaert R, Libert C (2021). Bidirectional crosstalk between hypoxia inducible factors and glucocorticoid signalling in health and disease. Front Immunol.

[77] Wang Y, Wan C, Deng L (2007). The hypoxia-inducible factor alpha pathway couples angiogenesis to osteogenesis during skeletal development. J Clin Invest.

[78] Lane NE, Mohan G, Yao W (2018). Prevalence of glucocorticoid induced osteonecrosis in the mouse is not affected by treatments that maintain bone vascularity. Bone Rep.

[79] Yu Haiyang LU, Zengpeng WANGH (2019). Experimental study on the changes of Hif-1α/VEGF signal axis and H-type vessels in hormone-induced femoral head necrosis. Chin J Exp Anim Sci.

[80] Li S, Zhan X, Zhang Y, et al. Effect of high dose glucocorticoid on PDGF-BB secretion and H-type vascular growth in adult male mice. Chin J Osteoporos. 27(12):1752–6.

[81] Peng Y, Lv S, Li Y (2020). Glucocorticoids disrupt skeletal angiogenesis through trans repression of NF-κB-Mediated preosteoclast Pdgfb transcription in young mice. J Bone Miner Res.

[82] Shangguan Y, Wu Z, Xie X (2021). Low-activity programming of the PDGFRβ/FAK pathway mediates H-type vessel dysplasia and high susceptibility to osteoporosis in female offspring rats after prenatal dexamethasone exposure. Biochem Pharmacol.

[83] Yang P, Lv S, Wang Y (2018). Preservation of type H vessels and osteoblasts by enhanced preosteoclast platelet-derived growth factor type BB attenuates glucocorticoid-induced osteoporosis in growing mice. Bone.

[84] Wang Y (2018). Angiogenin/ribonuclease 5 is an EGFR ligand and a serum biomarker for erlotinib sensitivity in pancreatic cancer. Cancer Cell.

[85] Yu W (2017). Plexin-B2 mediates physiologic and pathologic functions of angiogenin. Cell.

[86] Bai R, Sun D, Chen M (2020). Myeloid cells protect intestinal epithelial barrier integrity through the angiogenin/plexin-B2 axis. EMBO J.

[87] Li S, Goncalves KA, Lyu B (2020). Chemo sensitization of prostate cancer stem cells in mice by angiogenin and plexin-B2 inhibitors. Commun Biol.

[88] Vettori A, Greenald D, Wilson GK (2017). Glucocorticoids promote Von Hippel Lindau degradation and Hif-1α stabilization. Proc Natl Acad Sci USA.

[89] Wagner AE, Huck G, Stiehl DP, Jelkmann W, Hellwig-Bürgel T (2008). Dexamethasone impairs hypoxia-inducible factor-1 function. Biochem Biophys Res Commun.

[90] Gaber T, Schellmann S, Erekul KB (2010). Macrophage migration inhibitory factor counterregulates dexamethasone-mediated suppression of hypoxia-inducible factor-1α function and differentially influences human CD4^+^ T cell proliferation under hypoxia. J Immunol.

[91] Liao Z, Jin Y, Chu Y (2022). Single-cell transcriptome analysis reveals aberrant stromal cells and heterogeneous endothelial cells in alcohol-induced osteonecrosis of the femoral head. Commun Biol.

